# BigFoot: Bayesian alignment and phylogenetic footprinting with MCMC

**DOI:** 10.1186/1471-2148-9-217

**Published:** 2009-08-28

**Authors:** Rahul Satija, Ádám Novák, István Miklós, Rune Lyngsø, Jotun Hein

**Affiliations:** 1 Department of Statistics, University of Oxford, 1 South Parks Road, OX1 3TG Oxford, UK; 2 Alfréd Rényi Institute of Mathematics, Hungarian Academy of Sciences, Reáltanoda u. 13-15, 1053 Budapest, Hungary

## Abstract

**Background:**

We have previously combined statistical alignment and phylogenetic footprinting to detect conserved functional elements without assuming a fixed alignment. Considering a probability-weighted distribution of alignments removes sensitivity to alignment errors, properly accommodates regions of alignment uncertainty, and increases the accuracy of functional element prediction. Our method utilized standard dynamic programming hidden markov model algorithms to analyze up to four sequences.

**Results:**

We present a novel approach, implemented in the software package **BigFoot**, for performing phylogenetic footprinting on greater numbers of sequences. We have developed a Markov chain Monte Carlo (MCMC) approach which samples both sequence alignments and locations of slowly evolving regions. We implement our method as an extension of the existing StatAlign software package and test it on well-annotated regions controlling the expression of the even-skipped gene in *Drosophila *and the *α*-globin gene in vertebrates. The results exhibit how adding additional sequences to the analysis has the potential to improve the accuracy of functional predictions, and demonstrate how BigFoot outperforms existing alignment-based phylogenetic footprinting techniques.

**Conclusion:**

BigFoot extends a combined alignment and phylogenetic footprinting approach to analyze larger amounts of sequence data using MCMC. Our approach is robust to alignment error and uncertainty and can be applied to a variety of biological datasets. The source code and documentation are publicly available for download from

## Background

The identification of conserved DNA sequences by comparative genome sequence analysis has been widely used to annotate both protein-coding and gene regulatory elements in a wide variety of taxa [1-5]. While searching for these "phylogenetic footprints" [6] is a powerful technique, traditional methods often make predictions from a single DNA sequence alignment. By ignoring the possibility of alignment uncertainty, these predictions are highly sensitive to both alignment errors and regions where alternate alignments may describe the true evolutionary history. A dependence on a single alignment may be particularly harmful when searching for regulatory motifs, such as transcription factor binding sites (TFBS), which are difficult to align reliably due to their short lengths (6-15 nucleotides) and tolerance of degenerate nucleotides [7]. Recent studies have noted that single-alignment phylogenetic footprinting approaches often produce inaccurate or inconsistent results depending on the alignment method used, and have called for new techniques capable of controlling for alignment error and uncertainty [1,8-11].

"Statistical alignment" [12] methods provide a framework for performing comparative genomic analyses while considering a probability-weighted distribution of alignments. Probabilistic models of evolutionary events (insertions, deletions, and substitutions) are used to calculate the likelihoods of different evolutionary histories and a probability-weighted distribution of sequence alignments. These models vary in complexity, ranging from the treatment of insertion and deletion events (indels) as single-nucleotide events [13] to the modeling of complex length distributions for indels [9,14,15], but all allow for evolutionary inference without assuming a single alignment. Incorporating alignment uncertainty information using statistical alignment can improve not only the accuracy of sequence alignment, but also the estimation of the parameters specifying the length and frequency of evolutionary events as well as the estimation of phylogenetic relationships between species [9,16-19].

Statistical alignment models can be modified to simultaneously align sequences and detect functional elements. By doubling the number of states in a hidden markov model (HMM) aligner in order to model both quickly evolving (neutral) and slowly evolving (functional) elements, we recently introduced SAPF (a statistical aligner and phylogenetic footprinter), a software package which analyzes a probability-weighted distribution of alignments in order to identify sequence elements that are evolving at a reduced rate [20]. Results on both simulated datasets and *Drosophila cis*-regulatory modules demonstrate how removing the traditional dependence on a single alignment increases the accuracy of functional element predictions. The improvement was most prominent when there was alignment ambiguity in functional regions due to binding sites that were not highly conserved. While SAPF is used to discover new motifs, the MORPH software package modifies a simple probabilistic aligner to detect and align instances of known motifs that have been previously characterized as position state weight matrices [21]. Here too, the authors report higher accuracy when examining all alignments between two species.

While these studies present strong evidence for the benefits of using statistical alignment to detect regulatory elements, they are limited in the amount of sequence data they can analyze. Both SAPF and MORPH use standard HMM algorithms [22] to compute likelihoods and posterior probabilities, and as the number of sequences under analysis increases, the number of states in the HMM increases exponentially. As a result, MORPH is restricted to pairwise alignments and SAPF can analyze only up to four sequences. While the potential benefit of adding more sequence data is highly dependent on the evolutionary distances between species in the dataset, recent simulation studies have demonstrated how greater numbers of species can increase the sensitivity and specificity of functional element recognition [11,23]. Additionally, [23] proposed the simple rule that for a given evolutionary distance, the number of genomes required to detect functional elements scales inversely with element length. Therefore, while two genomes may be sufficient for detecting long conserved exons, three to fifteen genomes may be needed to detect TFBS. The inability to analyze more than four sequences puts SAPF at a disadvantage relative to phastCons, the single-alignment based phylogenetic footprinter used to create the 28-genome conservation track in the University of California at Santa-Cruz (UCSC) genome browser [5,24].

Markov chain Monte Carlo sampling techniques [25,26]have been successfully applied to statistical alignment methods in order to expand the numbers of sequences that can be analyzed [17,18,27]. The StatAlign package - a Markov chain Monte Carlo (MCMC) sampler implemented in Java - samples alignment parameters, sequence alignments, tree branch lengths and tree topologies in order to infer both the alignment and the phylogenetic tree relating the input sequences [28]. The sampler places a statistical alignment model on each branch of the tree, and represents internal node sequences as a collection of gaps and Felsenstein wildcards [27]. To create BigFoot, we extend this package to perform phylogenetic footprinting as well. We alter the alignment framework to model both quickly and slowly evolving regions, and develop new MCMC transition kernels to infer the breakpoints between the slowly and quickly evolving regions.

## Results and Discussion

### Algorithm

#### Model Summary

While traditional alignment algorithms assume identical mutation rates throughout the sequence, we introduce an alternative evolutionary model allowing for rate heterogeneity by modeling the evolution of both quickly and slowly evolving regions. In our model, a two-state HMM emits a sequence of conserved (slow evolution) and non-conserved (fast evolution) states at the root of the tree. This defines an alternating series of conserved and non-conserved segments, allowing our model to represent both neutral sequences expected to exhibit higher mutation rates and functional sequences undergoing purifying selection. Each segment evolves independently along a phylogenetic tree according to a pairwise alignment model which allows for insertions, deletions, and substitutions on each branch of the tree. While the StatAlign package jointly estimates both the tree and the alignment, we condition the analysis on a user-inputted phylogenetic tree in order to estimate the alignment and locations of quickly and slowly evolving regions more efficiently.

Our model is a reformulation of the SAPF multiple HMM with two main differences. As in [5], we model the difference between fast and slow states by scaling down the branch lengths of the phylogenetic tree in slow states, reducing the evolutionary time - and thus the expected divergence - in these regions. While SAPF models slow states by modifying the rate parameters of the mutation models, BigFoot uses a branch scaling approach in order to support multiple substitution models with different numbers of parameters. Two different scaling factors, both of which are model parameters endowed with user-defined priors and constrained to be less than one, are used for substitution and indel events.

Another minor difference is that the BigFoot model does not allow for insertions at the exact boundaries of functional regions. This modification was necessary to create unambigious and reversible MCMC proposals, and is a biologically relevant modification since an insertion event in one sequence should not define the beginning (or end) of a conserved region.

#### Modeling molecular evolution

Our alignment model is expressed as a pairwise HMM transducer [29,30], a conditionally normalized HMM representing the evolution of an ancestral sequence into a descendent sequence, and is similar to the transducer model in [20]. More complete details describing the transducer are presented in the **Methods **section and supplementary sections S1.1 and S1.2 [see Additional file 1].

We base our transducer on the 1992 approach of Thorne, Kishino, and Felstenstein (TKF92) [14]. TKF92 models the birth and death of fragments with geometrically-distributed lengths in order to represent long indel events. Our model can be viewed as an extension of the TKF92 approach, allowing these fragments to exist in either quickly or slowly evolving regions. In our fast states, as in the TKF92 model, the length of indel fragments is modeled by a geometric distribution with the same expected length as a fragment of matched bases, resulting in an expectation of long indel events. In annotated functional regions, however, we noticed that most indel events were very short (1-3 bp). To represent this, we create a separate parameter to specify the expected lengths of indel events. Thus, we not only expect functional regions to have fewer indel events, we also predict that these events will be shorter.

We place a pairwise transducer on each branch of the phylogenetic tree in order to model the evolution from each ancestor to each descendent. Transducer theory [29,30] shows how the concatenation of these transducers results in a multiple HMM describing the evolution from the ancestral root node to all leaves in the tree. We place a separate HMM on the root sequence, allowing it to switch between emitting slow-evolving characters and fast-evolving characters with specific probabilities. This models fast and slow regions with geometric distributed lengths, set by model parameters.

One limitation of our approach is that our model does not allow for the creation or deletion of conserved regions along the tree. Since the annotation of fast or slow characters emitted by the root is conserved in the descendent sequences, the model cannot detect the loss or gain of binding sites. For this reason, when testing BigFoot we discarded sequences with long deletions from analysis.

The full likelihood of a tree is equivalent to the full emission probability of the multiple-HMM. Unfortunately, this likelihood cannot be calculated quickly, as the time complexity of the Forward algorithm for a multiple HMM grows exponentially with the number of sequences. Instead of direct computation via dynamic programming, we apply a Bayesian MCMC method with data augmentation.

#### Bayesian MCMC

All model parameter densities are estimated using MCMC sampling. Exponential priors with expectation 1 have been used as priors for insertion-deletion parameters in the alignment transducer and for all free parameters in substitution models provided with the software package. For parameters responsible for annotation (branch scalings and expected lengths for fast/slow regions, as described above), we allow the user to input either Beta or uniform priors on these parameters. This allows the user to tailor the analysis to their specific needs. For example, the user can set an informative prior to search for longer weakly conserved regions, or for very short and highly conserved regions. Alternatively, the user can set uninformative priors and allow the MCMC to estimate parameter distributions freely.

The joint posterior distribution of alignments, trees and evolutionary parameters forms a high dimensional and complex distribution from which efficient direct sampling is most likely impossible. Therefore, we applied Markov chain Monte Carlo to converge to this prescribed distribution. After convergence, samples from the Markov chain provide correlated samples from the posterior distribution.

The likelihood of a tree under the multiple HMM can only be easily calculated when we augment the tree with additional data known as *extended alignments*. This data contains information on how the observed sequences are aligned to ancestral sequences associated with internal nodes of the tree. We represent the unobserved ancestral sequences as a collection of gaps and Felsenstein wildcards in order to sum over all possible nucleotide values when calculating the total likelihood.

Our MCMC walks on the joint distribution of the extended alignments, locations of fast and slow regions, and model parameters. The random walk comprises the following components:

• Changing model parameters

• Changing extended alignment

• Shifting the boundary of an existing fast or slow region

• Creating a new (or deleting an old) fast or slow region

The first two types of moves are described in [31], and the last two are described in the **Methods **section. In each MCMC iteration, we apply a Metropolis-Hastings move to alter one of these components, and select moves with fixed probabilities that were chosen to enhance mixing.

#### Postprocessing the samples from the Markov chain

The Markov chain provides correlated samples from the posterior distribution of alignments, locations of fast and slow regions, and evolutionary parameters. To report posterior probabilities for phylogenetic footprinting predictions we take the approach of [4,5,20], collapsing our predictions onto one axis and reporting posterior probabilities for a single species. Our results thus represent the posterior probability of each nucleotide having been generated from a slow state. These probabilities are simply the proportion of samples in which each nucleotide appears inside the boundaries of a slow region.

Multiple sequence alignment samples can be summarized in several ways. Unlike other authors [17], we found the MAP (Maximum a Posteriori) alignment estimation drawn from MCMC samples to be very unstable, especially when there is autocorrelation between samples from the chain. We chose instead to estimate the MPD (Maximum Posterior Decoding) alignment [32], which maximizes the product of alignment column posterior probabilities. We found this estimation to be more stable as it allows the uncertainty in each alignment column to be assessed independently. We present a complete algorithm for calculating the MPD alignment in supplementary section S1.3 [see Additional file 1].

### Testing

**Figure 1:**
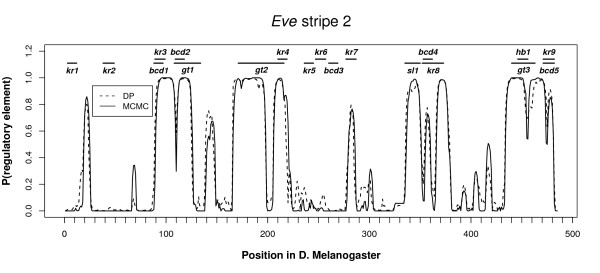
**Dynamic programming (SAPF) and MCMC (BigFoot) predictions along with annotated binding sites for the *eve *stripe 2 enhancer**. For each nucleotide in the *D. melanogaster *sequence, both programs output the probability that the nucleotide was generated by a functional (slow) state. Experimentally verified binding sites in *D. melanogaster *for the transcription factors, Bicoid (BC), Hunchback (HB), Kruppel (KR), Giant (GT), and Sloppy-paired 1 (Sl1) are shown above the posterior probabilities.

As a first test of the accuracy of the MCMC results, we ran BigFoot on a relatively small dataset to compare the results with the exact dynamic programming predictions of SAPF. The two methods were expected to return similar, though not identical, results. This is because SAPF and BigFoot use slightly different alignment HMMs on each tree branch. We analyzed a *cis*-regulatory module in four *Drosophila *species: *D. melanogaster*, *D. erecta*, *D. pseudoobscura*, and *D. willistoni*. This 485 base pair region has been found to regulate the expression of the homeodomain encoding protein *eve *in the second stripe of the developing *Drosophila *embryonic blastoderm [33]. The REDFly database provided the sequence coordinates of the biologically verified regulatory module in the *D. melanogaster *sequence [34], and the FlyReg database provided locations for 19 experimentally discovered binding sites [35]. Figure 1 exhibits the close agreement between the MCMC and dynamic programming predictions, with the locations of the known binding sites displayed above the posterior probabilities, and provides strong evidence that BigFoot is sampling from and converging to the true joint distribution. Both programs identify 14 of the 19 binding known binding sites with high posterior probabilities. Of the remaining five sites, none have homologous instances in either *D. melanogaster *or *D. pseudoobscura*. Four were biologically characterized as "weak-affinity" binding sites [36] which could indicate reduced functionality and a loss of evolutionary pressure, and the last was postulated to be recently evolved in *D. melanogaster *due to an absence of orthologous sequence in both closely and distantly related *Drosophila *species [33,37].

The *Drosophila *12 genome consortium has completed the sequencing of 12 *Drosophila *genome sequences exhibiting a large range of evolutionary distances [1,38]. For example, the evolutionary distance separating *D. melanogaster *and *D. grimshawi *is greater than that between any pair of mammals when generation time is taken into account [1], while other sequence pairs are very closely related. The large number of sequenced genomes and the diversity in their evolutionary distances make this an ideal dataset for implementing phylogenetic footprinting techniques. We tested the *eve *stripe 2 enhancer region using ten of the 12 genomes in this dataset. We chose to remove two species, *D. mojavensis *and *D. virilis*, as both sequences contained numerous long deletions and were thus too divergent to be informative. Sequence data for all species were obtained from a set of pre-computed whole-genome alignments [39].

**Figure 2:**
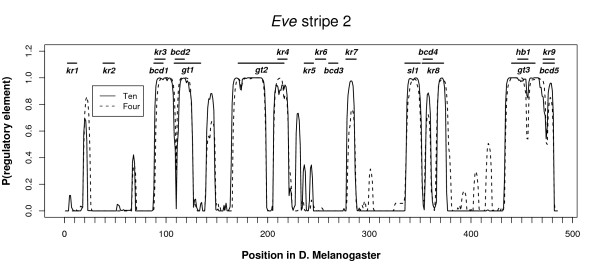
**BigFoot results for the *eve *stripe 2 enhancer when analyzing four sequences and ten sequences**. Increasing the number of species in the analysis results in higher posterior probabilities in many experimentally verified binding sites, and increases the nucleotide resoltion when identifying the precise locations for the TFBS.

Figure 2 compares MCMC predictions generated by BigFoot when analyzing either four sequences or ten sequences. The predictions made when analyzing the larger dataset correspond more closely to the locations of experimentally validated binding sites. The improvements can be summarized in two main categories:

**• Higher sensitivity to verified binding sites**. While Figure 2 exhibits a close agreement between the two sets of analyses for many of the experimentally verified binding sites, the addition of more species does improve the conservation signal in some TFBS. In particular, peaks corresponding to a Kruppel binding site (kr7), a Bicoid binding site (bcd4), and a joint site (kr9/bcd5) are all more strongly identified as evolving slowly when ten species are analyzed. Additionally, one Kruppel binding site (kr5) is only detected, albeit weakly, when using the larger dataset. This demonstrates that while imperfectly conserved regions may be reasonably likely to occur by chance in neutral sequence when only a few species are analyzed, additional sequences may provide stronger evidence of purifying selection. Heightened sensitivity is also observed at previously unannotated peaks from bases 137-148 and 227-232. Both regions are adjacent to a verified TFBS and have a high posterior probability of being emitted from a slow state, and thus should be candidate regions for future experimental study. This heightened sensitivity does not result in a general loss of specificity, as low probability peaks (bases 298-306 and 383-421) in previously un-annotated regions disappear when using the larger dataset.

**• Finer nucleotide resolution for TFBS start/stop positions**. When analyzing a small number of species, it may be difficult to identify the boundaries between quickly and slowly evolving regions, especially in a region where TFBS may be grouped close together. The results shown in Figure 2 demonstrate how adding additional sequence data can result in a clearer signal at the boundaries of binding sites. In the Kruppel site kr7, the distance in bases from the limits of the predicted conserved region (defined as the peak region with probability greater than 0.5) to the limits of the laboratory-identified regulatory element decreases by 3 bp when additional sequences are analyzed. Though this difference is small, it corresponds to 27% of the 11 bp binding site. A similar effect is observed in the Kruppel site kr8, for which additional sequence data decreases the boundary error by 4 bp.

Additionally, in a closely spaced group of functional elements (bases 440-482) separated only by a small number of neutral bases, the small dips in posterior probability correspond more closely to the neutral regions when more sequences are added to the analysis. While the agreement is not perfect, these results are consistent with previous findings showing an increase in nucleotide resolution as more species are analyzed [23].

**Figure 3:**
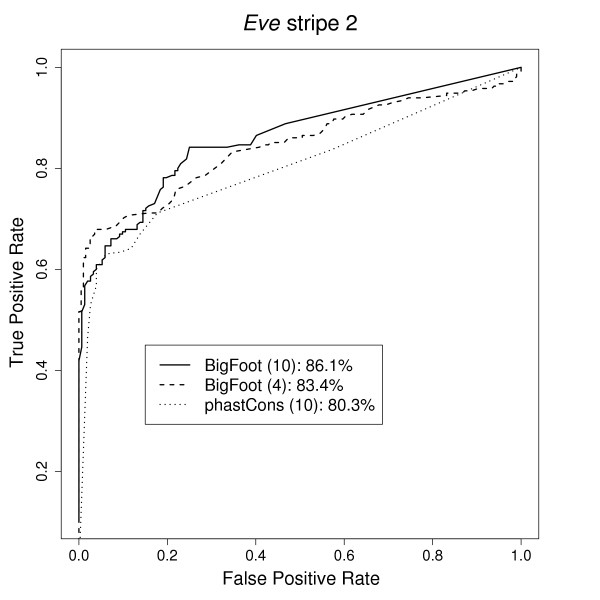
**ROC curves comparing the performance of BigFoot when analyzing four or ten sequences and phastCons analyzing ten sequences**. The figure legend shows AUC values for all curves. Among the three methods, optimal performance is acheived when BigFoot analyzes the larger dataset.

In order to quantify the predictive accuracy of our results, we calculated receiver operating characteristic (ROC) curves for both sets of BigFoot results. The area under the curve (AUC), which has a maximum value of 100%, is a summary statistic that accounts for both the sensitivity and the specificity of the predictions. A value of 50% implies that the predictions are no better than random guessing. The methodology used for creating the ROC curves is described in [20]. The curves are displayed in Figure 3, which exhibits the small but noticeable predictive improvement when additional sequences are added to the dataset.

To demonstrate that BigFoot can be applied to sequence data from vertebrates, we analyzed a 256 base pair region previously identified as the major regulatory element of human *α*-globin (*α*MRE) [40]. This region was sequenced in 22 species, analyzed with the TRANSFAC database, and found to contain seven TFBS. These TFBS range from 8-15 bp in length, and include recognition elements for the Maf protein and GATA-1, both important in globin gene regulation [40,41].

**Figure 4:**
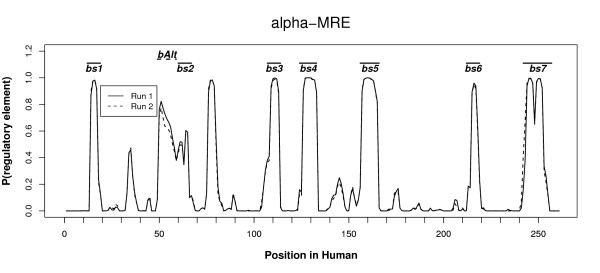
**Two independent BigFoot runs on the *α*MRE enhancer in 12 vertebrate species**. Despite having very different starting points, the two runs give essentially identical results, indicating convergence of the sampling distribution. The locations of seven previously identified binding sites are displayed above the posterior probabilities. The only binding site not detected with greater than 95% probability, bs2, is directly adjacent to a weakly conserved region (bsAlt) that is undetected by other methods due to alignment errors.

To analyze this region, we downloaded the multiz28way alignment of the region from the UCSC genome browser [42]. This alignment provided sequence information for 15 vertebrate species, three of which (cat, shrew, hedgehog) contained long deletions and were therefore removed from the analysis. The results of the analysis are shown in Figure 4, where we display the results of two independent MCMC runs initialized at independent starting points. The first run was started using the UCSC alignment to initialize the Markov chain, while the second was initialized with a random alignment proposed by BigFoot. Both runs were also initialized with independent and randomly selected evolutionary parameters. Despite these differences, Figure 4 exhibits that there is extremely close agreement - in many regions near exact correspondence - between the two sets of results, demonstrating the mixing and convergence of the MCMC sampling.

When analyzing the 12 vertebrate species, BigFoot detects six of the seven known binding sites with posterior probabilities of greater than 95%. However, the seventh binding site, notated in Figure 4 as bs2, is poorly conserved and the binding site peak probabilities do not exceed 50%. In fact, this region was only detected due to the presence of a previously unannotated and weakly conserved adjacent region, notated as bsAlt, which is incorrectly aligned in the multiz28 way alignment. BigFoot corrects this alignment error and annotates this region with a peak exceeding 80% posterior probability. This example demonstrates the importance of calculating and correcting for alignment ambiguity and error. By doing this, BigFoot not only discovers a previously undetected conserved region in a well-annotated regulatory module, but also enables the detection of a weakly conserved but previously identified regulatory element.

#### Comparison with phastCons

One of the most widely used alignment-based phylogenetic footprinting tools is the phastCons program, used to create the conservation track in the University of California at Santa-Cruz (UCSC) genome browser [5,24]. The conservation track makes predictions from a single multiz alignment and does not incorporate indel information. This puts the method at a significant disadvantage to BigFoot, as indel information has been shown to be extremely valuable in the detection of functional elements [43]. Indeed, we observed that predictions from the UCSC conservation track had significantly lower specificity and sensitivity when compared to the BigFoot results in both the *Drosophila *and the *α*MRE datasets.

In the command-line version of phastCons, however, it is possible to set an option for the program to incorporate indel information [44]. We set this option and ran phastCons on the BigFoot test datasets, using the UCSC alignment as input. The two programs returned almost identical results for the majority of the binding sites, since the majority of the binding sites are well conserved and thus perfectly aligned in the multiz alignments. In these cases there is little alignment uncertainty, so BigFoot and phastCons are expected to return similar results. However, in shorter binding sites exhibiting weaker conservation, BigFoot outperforms the single-alignment method. The most drastic example is the Kruppel site kr7 in the *eve *stripe 2 enhancer. The core of the TFBS is very well conserved and BigFoot predicts the site with high probability, but there are substitutions towards the edges and short indels in some species, and the multiz alignment incorrectly aligns the binding site. As a result, phastCons detects all nucleotides in the TFBS with less than 5% probability. The ROC curve for the phastCons predictions is displayed alongside the BigFoot ROC curves in Figure 3. Comparing the AUC values demonstrates how the increase in predictive accuracy caused by adding more sequence data is less than the corresponding increase caused by analyzing a distribution of alignments instead of a single alignment.

A similar error in the multiz alignment of the *α*MRE enhancer results in phastCons failing to annotate any nucleotide the weakly conserved region discussed in the previous section (bAlt) with greater than 5% probability. While we cannot know if this region is a functional TFBS without further experimental analysis, examining a distribution of alignments ensures that this region is not incorrectly passed over during the conservation analysis.

Since phastCons analyzes only one single alignment, the computational time to analyze a single region, around 30 seconds on a 2 ghz macbook computer, was substantially less than the 12-16 hours needed to analyze a single region with BigFoot. As a result, phastCons can be used to compute functional element predictions for the entire genome, while BigFoot can only be used to analyze individual regions of interest. However, for users who have identified specific genomic regions to study in detail, the benefit of controlling for alignment error and uncertainty by using BigFoot may justify the additional computational time needed for the analysis.

### Implementation

The algorithms have been implemented in Java 1.5, and are part of the BigFoot software package available at: 

#### User input

BigFoot requires the user to input a set of homologous DNA sequences (in FASTA format) and an evolutionary tree (in newick format) describing the phylogenetic relationships between the inputted species. BigFoot can construct an initial alignment of the sequences, or if the user has a previously computed starting alignment in FASTA format, they can set it as the starting alignment in the Markov chain. The user can also place either Beta or uniform priors on parameters modeling the difference between fast and slow states.

#### Substitution models

Our aim was to build a software package for an insertion-deletion model that can be coupled with an arbitrary substitution model. Therefore we would like to give users the option to implement their own substitution models. In the software help file, we describe how users can extend this class to create their own substitution models. We currently provide a large selection of eight nucleotide substitution models including the Jukes-Cantor model [45], the Kimura three parameter model [46], and the HKY85 model [47].

#### Postprocessing

Our program provides random samples from a Markov chain whose stationary distribution is the joint Bayesian distribution of sequence alignments, locations of fast and slow regions, and model parameters. This high dimensional joint distribution can be analyzed in several ways, ranging from an analysis of the posterior distribution of a single rate parameter to an investigation of Markov chain convergence using a log-likelihood trace or a separate multidimensional autocorrelation analysis. We implemented a set of postprocess plugins which analyze data from the Markov chain and display in the graphical interface. In the software help file, we also describe how the user can implement their own postprocess plugin by extending the abstract class.

**Figure 5:**
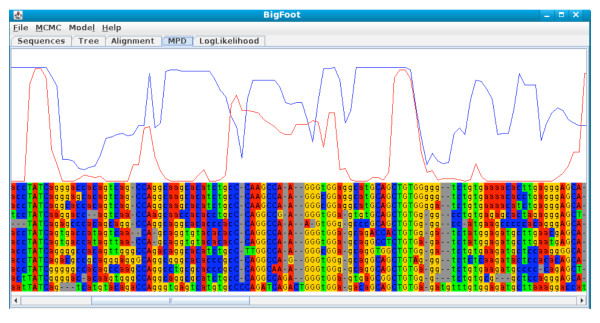
**BigFoot screenshot showing a part of the estimated Maximum Posterior Decoding alignment during an MCMC run**. This screenshot is taken from an analysis of the *α*MRE enhancer region using 12 vertebrate species. The blue curve represents BigFoot's confidence in the correctness of the alignment, and the red curve represents the phylogenetic footprinting predictions.

We implemented the following plugins, each of which represents a tab in the graphical interface:

• **Log-likelihood trace **This plugin plots the log-likelihood trace and writes the log-likelihood values into a text file when the analysis is complete.

• **Current alignment **This plugin shows the multiple sequence alignment in the current state of the Markov chain, along with the locations of slow and fast regions. Capital letters in the alignment represent slowly evolving regions in the current state of the chain, while lower case letters represent quickly evolving regions.

• **MPD alignment **This plugin calculates and performs running updates of the Maximum Posterior Decoding alignment based on all previous samples of the Markov chain. Figure 5 pictures this alignment display, along with two curves overlaying the sequence information. The blue curve represents the posterior probability of each alignment column: higher values indicate greater confidence in the alignment. The red curve represents the phylogenetic footprinting results: higher values indicate a greater posterior probability of purifying selection. The final MPD alignment, and all footprinting results, are written into a text file after the analysis is complete.

• **Current tree **This plugin graphically displays the tree inputted by the user.

### Computational power

We initially assessed convergence using a log-likelihood trace, and verified convergence using independent MCMC runs. We found that 10^6^-10^7 ^steps were required for convergence, depending on the number of sequences and their lengths. To remove the effects of autocorrelated samples, we took a sample of the chain after every 5000 iterations of the MCMC. For all examples, total computational time did not exceed 16 hours on a 2 ghz Macbook computer. Datasets larger than 12 species can also be analyzed, but may take longer to achieve convergence.

## Conclusion

We have presented and tested an algorithm for co-sampling multiple sequence alignments, locations of quickly and slowly evolving regions, and a set of evolutionary parameters. Our likelihood engine evaluates an HMM transducer switching between fast and slow states, where the evolutionary models in the slow states indicate a reduced rate of mutation as a consequence of purifying selection. We also present a new MCMC transition kernel enabling the combination of sequence alignment and phylogenetic footprinting. We have demonstrated the accuracy of our method by comparing the results with a dynamic programming solution, and we have presented strong evidence for the convergence of our sampling distribution by running independent MCMC runs from different starting points and obtaining essentially identical results.

We tested BigFoot on the *eve *stripe 2 gene in *Drosophila*. Our results exhibit two major potential benefits for analyzing additional sequences in comparative genomics approaches. Augmenting our dataset from four to ten sequences resulted in higher sensitivity towards experimentally verified binding sites and finer nucleotide resolution when detecting the exact boundaries for TFBS. The ability to analyze larger datasets was the primary motivation for extending the SAPF dynamic programming approach into BigFoot, and these results show that it is important for existing tools to have the capacity to analyze multiple sequence datasets.

By simultaneously estimating multiple sequence alignments and phylogenetic footprinting predictions, BigFoot correctly incorporates alignment uncertainty information into functional element predictions, and ensures that alignment error or ambiguity will not prevent the software from identifying slowly evolving regions. However, there is an additional benefit to our joint model. In some weakly conserved regions there is a highly conserved core of 3-5 bp, but conservation drops off slightly towards the edges of the site. In these cases, a simple aligner will often correctly align only the binding site core while misaligning the outer regions, or misalign the entire binding site. Our combined model, however, recognizes that a conserved core may indicate the presence of a slowly evolving region, and thus could be surrounded by other conserved nucleotides. In these cases, the model will push weakly conserved positions together to align the full binding site. As a result, BigFoot not only detects the binding site, but also increases the accuracy of the posterior alignment distribution. The two best examples of this phenomenon are the two conserved regions (the bsAlt region in vertebrates and a Kruppel site in *Drosophila*) that were misaligned in the multiz alignments. While both these regions contained small indel events and multiple nucleotide degenerate sites, resulting in multiple plausible evolutionary histories, the posterior alignment distribution from BigFoot exhibited how our joint model reliably aligned all instances of the binding site together.

While there are binding sites for which analyzing a distribution of alignments improves the accuracy of BigFoot's predictions, for many other binding sites, analyzing a single alignment may return very similar results. The latter case is particularly true when binding sites are highly or perfectly conserved in which case the bulk of the probability mass in the alignment distribution rests on a single alignment. Thus, if all binding sites in a region are highly conserved, the approximation of a single alignment is adequate and BigFoot may not significantly improve upon traditional methods. When roughly analyzing large genomic regions or large numbers of regulatory modules, traditional methods like phastCons may correctly predict the majority of binding sites. The additional computational time and complexity required to calculate the alignment distribution may reduce BigFoot's practicality for these datasets. However, during detailed analysis of individual regions, such as identifying specific sites for further laboratory analysis, the potential for BigFoot to correct for alignment error and uncertainty may justify the neccessary additional computational time. We are currently exploring different techniques for drastically reducing BigFoot's computational requirements. For example, approximating the multiple alignment distribution by analyzing the set of all pairwise alignments instead of using MCMC could allow for the analysis of large genomic regions.

Another particularly useful improvement to our model would be to relax the constraint fixing the phylogenetic footprinting annotation of all species in an alignment column. This would allow us to appropriately model the gain and loss of functional regions in parts of the tree. We are currently pursuing this improvement, hoping that it will not only improve our ability to detect weakly conserved binding sites but will also allow us to make statistical predictions about the evolution of regulatory elements in a species or clade.

## Methods

### The Alignment Transducer

**Figure 6:**
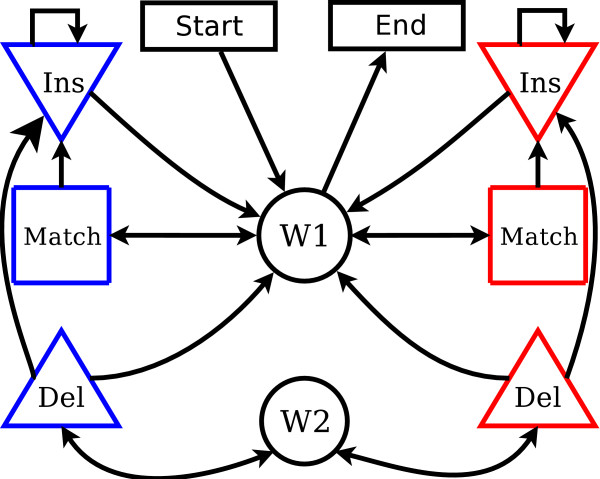
**The alignment transducer used to model evolution from the ancestor to the descendent on each branch of the phylogenetic tree**. Match, Insert (Ins), and Delete (Del) states represent evolutionary events. States with blue outlines (slow states) have reduced rates of evolution compared to states with red outlines (fast states). The model enters one of the wait states (W1 and W2) when waiting for input from the ancestral sequence.

Our model is powered by an evolutionary transducer describing the evolution between the ancestor and the descendent on each branch of the phylogenetic tree. This transducer, shown in Figure 6, models the evolutionary history of insertions and deletions in order to infer a DNA sequence alignment, but also identifies slowly evolving regions represented by three slow states (colored in blue). As described previously, evolutionary time is scaled down in slow states to represent the effects of purifying selection.

An HMM transducer is similar to a pairwise HMM. However, all transitions and emissions in a transducer are normalized conditional on the input (ancestral) sequence. Transitions to a slow state only occur when there is a "slow" character emitted in the ancestral sequence, and the same is true of fast states and fast characters. The model switches between fast and slow states only when a character type switch is emitted from the ancestral root. As previously discussed, the ancestral root node switches between emitting fast and slow characters according to a basic hidden markov model.

The transducer model contains two "wait" states, W1 and W2, to which the model transitions while waiting for input from the ancestral sequence. These transitions occur after the transducer has finished processing the previous character from the ancestral sequence (for example, after the ancestral character has been either matched or deleted in the descendent), and the transducer will remain in the wait state until the ancestral sequence emits the next character. This allows the transducer to pause while other evolutionary events, such as indels on a different half of the tree, occur in other sequences. A second wait state is needed since unlike the insertion state, the delete state cannot self-transition. This second wait state, which only the delete state can access, creates an effective self-transition that allows for the modeling of long deletion events. The full transition matrix for our HMM transducer is shown in supplementary section S1.2 [see Additional File 1] and a complete explanation of wait states and the general application of HMM transducers to sequence alignment can be found in [29].

### MCMC Transition Kernels

**Figure 7:**
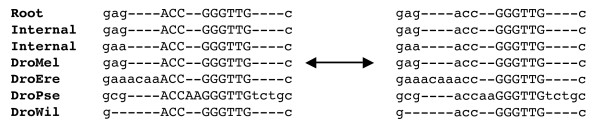
**MCMC move where a boundary between slow and fast regions is shifted by three root nucleotides**. This move is especially important in accurately calculating footprinting posterior probabilities at the edges of functional regions. We do not allow insertions to occur at the beginnings or ends of functional regions, as shown in the alignment on the right. For display purposes, nucleotides emitted from slowly evolving regions are represented as capital letters. While we store internal sequences as Felstenstein wildcards, we display the most likely nucleotide at each position here.

In order to combine both alignment and phylogenetic footprinting, we needed to introduce new sampling moves for our random walk. At each state in the chain, we kept track of the points where the HMM transducer switched between fast and slow states, corresponding to a predictive switch between neutral sequences and functional elements. For display purposes (see Figure 7), we capitalized nucleotides emitted from slow states in the sequence alignment at each step. We found that only three new types of moves were necessary to provide good mixing.

**Figure 8:**
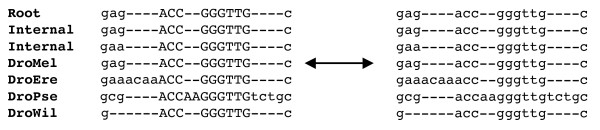
**MCMC move creating a new pair (when going from right to left) or deleting an existing pair (going from left to right) of boundaries**. To improve mixing and convergence, we weight our proposals based on the lengths of the new or existing regions and their approximate level of conservation.

• **Shifting location of existing boundaries**. We extend (or shrink) an existing boundary and calculate the effect on the overall likelihood. We fix the root sequence and switch a small number of nucleotides (determined by a geometric distribution) in the root between fast and slow. Figure 7 displays a boundary shift of three nucleotides. This move is especially helpful for accurately calculating footprinting posterior probabilities near the ends of functional regions.

• **Creating a new pair of boundaries**. This move proposes either a new slowly evolving region in a stretch of neutral sequence (see Figure 8), or a new neutral region in the middle of a conserved element, creating two separate binding sites. The lengths of the new regions are proposed from simple stepwise distributions. When proposing new conserved regions, we expect the lengths to span approximately 5-10 bp. We propose shorter lengths for new quickly evolving regions when splitting a conserved region in two, as there are often short stretches of 1-4 degenerate nucleotides in a long conserved binding site.

To achieve better mixing, we scan the existing alignment for areas where new boundaries may improve the likelihood before proposing new regions. We calculate a very basic conservation score, based on the number of mutations in the alignment column, and weight our proposals towards regions with high conservation (for new slowly evolving regions) or low conservation (for new quickly evolving regions).

• **Deleting an existing pair of boundaries **This move is the exact reverse of the move described above, and is also pictured in Figure 8. It corresponds to the merging of three heterogeneous regions into one homogeneous region. When proposing regions to merge, we weight our proposals towards regions whose lengths differ significantly from the expected geometric distribution set by the HMM in the ancestral root.

After each of these moves, we calculate the probability of proposing the new state and the probability of back-proposing the old state, along with the resulting change in likelihood. We then accept or reject the move, with an acceptance probability set by the Metropolis-Hastings ratio.

## Availability and Requirements

• Project name: BigFoot

• Project webpage: 

• Operating system: Platform independent

• Programming language: Java

• Other requirements: Java Virtual Machine 1.5 or higher

• License: GNU GPL

## Authors' contributions

RS wrote the majority of the phylogenetic footprinting code and performed the biological dataset analysis. IM, AN, and RL wrote the alignment software and postprocessing code. All authors contributed to the manuscript. JH encouraged the research and supervised the project.

## Supplementary Material

Additional file 1 **Technical Methods**. Additional technical information describing the BigFoot transducer parameters and the algorithm used to estimate MPD alignments on-the-fly. Click here for file
