# Supplementary material for: BigFoot: Bayesian alignment and phylogenetic footprinting with MCMC
Source: BMC Evol Biol. 2009 Aug 28;9:217. doi: 10.1186/1471-2148-9-217 (PMC2744684; doi:10.1186/1471-2148-9-217)
Supplement: Additional file 1 — Technical Methods. Additional technical information describing the BigFoot transducer parameters and the algorithm used to estimate MPD alignments on-the-fly. [file 1471-2148-9-217-S1.pdf]

1 Technical Methods Description

S1.1 Parameters

Table 1 summarizes all model parameters used in BigFoot. All parameter distributions are estimated in the MCMC sampling, and any prior distributions are detailed in the manuscript.

Table 1: BigFoot Parameters

Parameters	Description
λ	Birth rates for fragments.
μ	Death rates for fragments.
R	Geometric distribution parameter for fragment length.
σ	Set of parameters for user-chosen substitution model.
α	Branch scaling factor for substitutions in slow regions.
β	Branch scaling factor for indels in slow regions.
δ	Root HMM self-transition probability for staying in a fast state.
ϵ	Root HMM self-transition probability for staying in a slow state.
τ	Indel self-transition probability in slowly evolving regions.

S1.2 Transition Probabilities

Eqns. 1 and 2 display a full transition matrix for the fast and slow states of the BigFoot model. All transitions in an HMM transducer are conditionally normalized on an input sequence, and a transition to a fast state can only occur if a fast character is emitted from the root HMM. This is why we split the overall transition matrix into two separate equations.

When the root HMM switches between emitting fast symbols and slow symbols, the transducers on each branch of the tree must make an intermediate transition the W1 state, which connects the fast and slow states together. For example, suppose the transitioning between an alignment column of all matched characters in a fast region, and an alignment column of all matched characters in a slow region. In this case, the transducer on each branch must first transition from the M_{fast} state to W1, and then immediately transition from W1 to M_{slow}. Eqns. 1 and 2 are very similar, with two exceptiosn. In slow states, the evolutionary time t, separating the ancestral and descendent sequences, is multiplied by a scaling factor β to model the effects of purifying selection. Additionally, the

$$
\begin{array}{c} \\ S \\ M \\ I \\ D \\ W1 \\ W2 \end{array}
\begin{array}{c}
\begin{array}{ccccccc} S & M & I & D & W1 & W2 & E \end{array} \\
\begin{pmatrix}
0 & 0 & \lambda\zeta(t)\frac{\lambda}{\mu} & 0 & (1-\lambda\zeta(t))\frac{\lambda}{\mu} & 0 & 1-\frac{\lambda}{\mu} \\
0 & 0 & (1-R)\lambda\zeta(t) & 0 & R+(1-R)(1-\lambda\zeta(t)) & 0 & 0 \\
0 & 0 & R & 0 & 1-R & 0 & 0 \\
0 & 0 & (1-R)(1-\frac{\lambda\zeta(t)}{1-e^{-\mu t}}) & 0 & (1-R)\frac{\lambda\zeta(t)}{1-e^{-\mu t}} & R & 0 \\
0 & e^{-\mu t}\frac{\lambda}{\mu} & 0 & (1-e^{-\mu t})\frac{\lambda}{\mu} & 0 & 0 & 1-\frac{\lambda}{\mu} \\
0 & 0 & 0 & 1 & 0 & 0 & 0
\end{pmatrix}
\end{array}
\quad (1)
$$

Eqn. 1 : Transducer transition matrix for fast states. All transitions are conditioned on the emission of a fast character in the ancestral sequence.

$$
\begin{array}{c} \\ S \\ M \\ I \\ D \\ W1 \\ W2 \end{array}
\begin{array}{c}
\begin{array}{ccccccc} S & M & I & D & W1 & W2 & E \end{array} \\
\begin{pmatrix}
0 & 0 & \lambda\zeta(\beta t)\frac{\lambda}{\mu} & 0 & (1-\lambda\zeta(\beta t))\frac{\lambda}{\mu} & 0 & 1-\frac{\lambda}{\mu} \\
0 & 0 & (1-R)\lambda\zeta(\beta t) & 0 & R+(1-R)(1-\lambda\zeta(\beta t)) & 0 & 0 \\
0 & 0 & \tau & 0 & 1-\tau & 0 & 0 \\
0 & 0 & (1-\tau)(1-\frac{\lambda\zeta(\beta t)}{1-e^{-\mu\beta t}}) & 0 & (1-\tau)\frac{\lambda\zeta(\beta t)}{1-e^{-\mu\beta t}} & \tau & 0 \\
0 & e^{-\mu\beta t}\frac{\lambda}{\mu} & 0 & (1-e^{-\mu\beta t})\frac{\lambda}{\mu} & 0 & 0 & 1-\frac{\lambda}{\mu} \\
0 & 0 & 0 & 1 & 0 & 0 & 0
\end{pmatrix}
\end{array}
\quad (2)
$$

Eqn. 2 : Transducer transition matrix for slow states. All transitions are conditioned on the emission of a slow character in the ancestral sequence.

transitions out of insertion and deletion states have been modified to reflect the observed shorter length distributions of indels in functional conserved sequence.

To further simplify the notation below, we define $\zeta(\tau) = \frac{1-e^{\lambda-\mu)\tau}}{\mu-\lambda e^{\lambda-\mu)\tau}}$

S1.3 Fast on-the-fly estimation of Maximum Posterior Decoding alignments

The Markov chain provides correlated samples from the posterior distribution of alignments, trees and evolutionary parameters. These samples can be summarised in several ways. We found the MAP (Maximum a Posteriori) alignment estimation drawn from MCMC samples very unstable. The reason behind it is that there might be several local uncertainties in a multiple alignment. Any combination of these locally alternative alignments yields a likely global alignment. If the number of regions suffering from alignment uncertainty is high, then the number of likely global alignments will be very high. The number of samples from the Markov chain has to be at least two orders of magnitude larger – when autocorrelation is high, even more – than the number of alignments contributing significantly to the posterior distribution to get a reasonable estimation on

what is the most likely alignment in the posterior distribution. If there are 4-6 regions in the multiple alignment where there are 5-10 likely local alignments, then the number of samples from the Markov chain has to be at least 10^6-10^8 to get a good covering of the posterior distribution of alignments. Given that there has to be at least 10^3-10^4 steps between two samples in the Markov chain to get low autocorrelation between samples, and one MCMC step takes about 0.01-0.1 second on a modern computer when aligning 6-10 sequences, 200-300 characters each, it would take years to generate this number of samples.

Instead of MAP alignment estimation, we estimate MPD (Maximum Posterior Decoding) alignments [1] from the Markov chain. The MPD alignment is the alignment that maximises the product of posterior probabilities of alignment columns. We use the sampling frequencies of multiple alignment columns in the Markov chain as the estimator for the posterior probabilities. We create the network of all multiple alignment columns that appear in the samples from the Markov chain, and we apply simple dynamic programming to get an estimation for the MPD alignment.. This estimation is more stable since each multiple alignment provides samples on all of its alignment columns, and these samples are processed independently in the dynamic programming algorithm that estimates the MPD alignment. Hence, each local uncertainty can be decided independently of the other local uncertainties.Our algorithm for calculating the MPD alignment and updating it on-the-fly is described below.

Preliminaries

Sequences will be denoted by S_1, $S_2, \ldots S_n$, and the length of sequence S_i will be denoted by l_i. We use $s_{i,j}$ to refer to the character at position j in sequence S_i, where $1 \leq j \leq l_i$. A multiple alignment A of n sequences is an $n \times m$ table where entry $a_{i,j}$ is either a gap symbol $(-)$ or a character in sequence S_i. The non-gap characters on line i of A are the characters of sequence S_i in the same order.

We will decode a multiple alignment into a code table C as follows. If $a_{i,j} = s_{i,k}$ then let $c_{i,j} = 2k-1$. Otherwise (if $a_{i,j}$ is a gap), $c_{i,j}$ will be an even number, the exact value depending on the previous non-gap character on line i. If there is no non-gap character on line i before $a_{i,j}$, then $c_{i,j} = 0$, otherwise if the last non-gap character on line i before $a_{i,j}$ is $s_{i,k}$, then $c_{i,j} = 2k$.

The jth alignment column is a vector containing characters $a_{\cdot,j}$, its code vector contains codes $c_{\cdot,j}$. Two alignment columns from two different alignments are considered identical if and only if they share the same code vector. For example, the `-/-/A` columns in the following two alignments are distinguished since their code vectors (4,4,5) and (2,2,5) differ:

```
AC-C-    A--CC
AC-C-    A--CC
ACACC    ACACC
```

Given a set of alignments, their alignment columns form a directed acyclic graph in the following way. Each alignment column is transformed into the

corresponding code vector, and the copy number of each code vector in the set of alignments is counted. We further add a start and an end code vector, with the start vector containing all -1s and the end vector containing $2l_i + 1$ at position i. The nodes of the DAG will be the alignment columns represented by their code vectors together with the start and the end code vectors. There is an edge from node v to node w if and only if for all coordinates i of their corresponding code vectors $\mathbf{v}$ and $\mathbf{w}$ either both v_i and w_i are even and $v_i = w_i$ or at least one of them is an odd number and $1 \leq w_i - v_i \leq 2$. Since the values in any coordinate cannot decrease along any directed path, the graph will obviously be acyclic. It is easy to see that any path from the start node to the end node gives a valid multiple alignment, since each line in the obtained alignment has to contain all characters from the corresponding sequence in the appropriate order. Indeed, in the corresponding code table, $c_{i,j} = 2k$ can be followed by either $c_{i+1,j} = 2k$ or $c_{i+1,j} = 2k+1$ and $c_{i,j} = 2k+1$ can be followed by either $c_{i+1,j} = 2k + 2$ or $c_{i+1,j} = 2k + 3$.

Let v_f denote the frequency of the alignment column associated to node v. The estimation of the MPD alignment given a set of alignments is the path π that maximises

$$\sum_{v \in \pi} v_f \tag{3}$$

This path can be found by standard dynamic programming [2].

Since we use this DAG for MPD estimation, we will refer to this DAG as MPD-DAG to distinguish it from another DAG introduced in this paper.

Fast addition of sampled alignments

When the multiple alignments are sampled from a Markov chain Monte Carlo algorithm, a new multiple alignment has to be "threaded" into the already constructed MPD-DAG. It means that for each of its alignment columns, it has to be decided whether or not the column is already represented in the graph. If so, then its frequency has to be increased. If not, then all of its preceding and following vertices have to be found and connected to the new vertex. The naïve algorithm visits each vertex and checks if it is a preceding or following vertex of the vertex to be added, and hence the worst case running time for threading a new alignment into the MPD-DAG is $\Omega(|A||D|)$ where $|A|$ is the size of the multiple alignment and $|D|$ is the size of the MPD-DAG.

Inspired by the classic "forward-looking" dynamic programming [3] introduced in the A^* algorithm [4], a faster method would maintain a candidate list of potential preceding vertices and might have a better running time. However, an even faster method is available that we describe below.

Correspondence between the DP-DAG and MPD-DAG

Our observation is that the MPD-DAG we are constructing is a sparse subgraph of a graph that is related to the DAG represented in the standard dy-

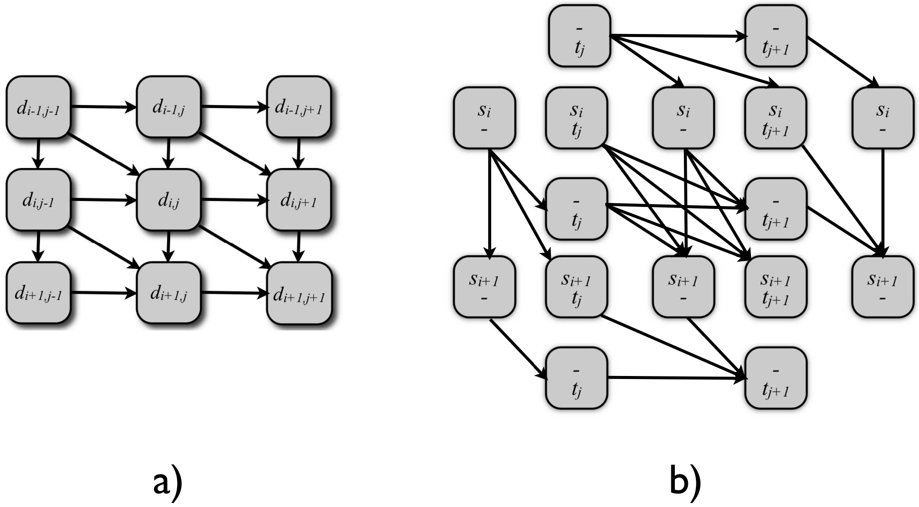

Figure 1: Relationship between the dynamic programming directed acyclic graph (DP-DAG, **a)**) and the MPD directed acyclic graph (MPD-DAG, **b)**). The vertices of the MPD-DAG are the edges of the MP-DAG, and two vertices are connected in the MPD-DAG if the target of one of the corresponding edges in the DP-DAG equals the source of the other.

namic programming table for calculating the optimal multiple alignment that we will hence call DP-DAG. This correspondence in the two dimensional case is illustrated in Fig. 1, the generalisation for multiple sequences and dimensions is obvious. The standard dynamic programming table for two sequences of length n and m, respectively, is a $(n+1)\times(m+1)$ table T, with indices starting from 0. $t_{i,j}$ tells the score of the optimal alignment between the prefixes of length i and j, with prefixes of length 0 being the empty sequence. The dynamic programming recursion calculates $t_{i,j}$ using $t_{i-1,j}$, $t_{i,j-1}$ and $t_{i-1,j-1}$. This recursion can be represented by a directed acyclic graph whose vertices are the entries in T and there is an edge connecting v to w iff v is used in calculating w. Each edge can be a horizontal, a vertical or a diagonal step, and represents an insertion, deletion or match, respectively. Hence, these edges represent the possible alignment columns that are the vertices of an MPD-DAG. Therefore, there is a one-to-one correspondence between the edges of a DP-DAG and the vertices of the MPD-DAG, and two vertices are connected in the MPD-DAG if and only if the corresponding edges in the DP-DAG are neighbours.

Hashing and bagging vertices of the MPD-DAG

This observation immediately gives an efficient algorithm for threading incoming multiple alignments into an MPD-DAG. We construct three hash maps. The first is called column hash map, and it contains alignment columns, i.e. vertices

of the MPD-DAG. The hash key is the corresponding code vector. The other two hash maps contain bags, each bag is an array of alignment columns. We call these hash maps start-bagging and end-bagging hash maps. Each bag in the start-bagging hash map contains alignment columns that have the same starting point in the corresponding DP-DAG. That is, each bag corresponds to an entry in the dynamic programming table. The bags in the end-bagging hash map are also related to the entries in the dynamic programming table, they contain the alignment columns of the MPD-DAG whose corresponding edges in the DP-DAG have the same end points. The hash key of the bagging hash maps are the indices vector of their corresponding entry in the dynamic programming table.

The fast threading method now works as follows. For each alignment column c in the incoming multiple alignment, the algorithm calculates the corresponding code vector, as hash key, and asks the column hash map if c is already represented in the MPD-DAG. If this is the case, then it returns a reference to c and the frequency of the alignment column in the MPD-DAG is updated.

Otherwise, c is added both to the column hash map and to the MPD-DAG. Then the algorithm calculates the hash key c_{start} of c in the start-bagging hash map, and adds c to this bag (which may have to be created if it was previously empty). All columns that can precede c in an alignment corresponds to edges that end at the entry in the dynamic programming table where the edge corresponding to c begins. Hence, all valid preceding columns that have been encountered so far will be in the end-bag with key c_{start}, so we add an edge from each of these to c in the MPD-DAG. Similarly, the algorithm calculates the hash key c_{end} of c in the end-bagging hash map, adds c to this bag (which also may have to be created), and connects c to all encountered columns that can validly follow c in an alignment which are available in the start-bag with key c_{end}. The full procedure is formalised in Alg. 1. It is a trivial observation that the total time required to maintain the MPD-DAG using this procedure is $O(|D_E| + ns)$, where D_E is the set of edges in the final MPD-DAG, n the number of sequences, and s the total number of sampled alignment columns. Observe that this is linear in the sum of the sizes of the input and the output, and hence optimal.

Fast update of the MPD alignment estimation

Once the incoming alignment is threaded into the MPD-DAG, the MPD alignment estimation can be updated. The "forward looking" algorithm [3] visits only the nodes whose dynamic programming score changes. The frequencies on the path representing the new alignment changed, but the values used in the dynamic programming (namely, the sum of the frequencies for the best sub-path ending in the given vertex) might change at other vertices in the MPD-DAG, too. Indeed, the dynamic programming score might change for a vertex if it is changed in any of its preceding vertices.

The forward-looking dynamic programming does not select the best candidate score calculated from the score of the preceding vertices of the actual

Algorithm 1 Algorithm for updating MPD-DAG D with alignment column c represented by its code vector. H denotes the column hash map, S the start-bagging hash map, and E the end-bagging hash map, and **div** is component-wise integer division.

```
if c ∈ H then
   Increment count of c in D
else
   Add vertex c to D with unity count and insert c in H
   Calculate c_start = c div 2 and add c to S[c_start]
   for v ∈ E[c_start] do
      Add edge (v, c) to D
   Calculate c_end = (c + 1) div 2 and add c to E[c_end]
   for v ∈ S[c_end] do
      Add edge (c, v) to D
```

vertex, but sends ahead candidate scores to the following vertices of the actual vertex. This strategy is particularly useful when one would like to update the MPD alignment in the MPD-DAG after threading a new alignment into the MPD-DAG. Indeed, if the sent-ahead candidate value is worse than the actual value in a following vertex, then this following vertex does not have to send ahead candidate values. Therefore the fast update method first makes a priority queue from the threaded alignment columns, then visits the elements in the priority queue. All visited alignment columns send ahead candidate scores, and if a candidate score is better than the actual score in a following alignment column, then the score of this alignment column is updated and the column is added to the priority queue. If the queue is ordered by the lexicographic order of the code vectors, then it is guaranteed that a vertex has the proper dynamic programming score when it sends ahead candidate values.

We used the built-in optimised HashMap class. Its public `get(Object key)` function returns the hash map element with the specified key in $O(1)$ amortised time. This implementation (with the default load factor) is efficient enough for our purposes and we relied on it for the on-the-fly monitoring of the current estimation of the MPD alignment.

The forward-looking dynamic programing algorithm for updating the MPD alignment estimation did not yield significant improvements in practice compared to the backward-looking algorithm that visits all the nodes in the MPD-DAG. Detailed investigation revealed that the MPD alignment estimation might change significantly when only a few samples are available from the Markov chain. Hence, the forward-looking algorithm also visits the majority of the nodes in the MPD-DAG, and moreover, all updated nodes have to be inserted into the priority queue, which takes time proportional to the logarithm of the queue length. As the sampled alignments accumulate, the MPD alignment estimation becomes more stable, and the performance of the forward-looking algorithm slightly improves. However, even if the MPD alignment does not change, the

scores in other alignment columns change, and hence, the forward-looking algorithm still visits many nodes. The reasoning for this is the following. Assume that the first column in the incoming alignment is a very frequently observed column, i.e. it has a high score. Then it will be the best beginning to almost all MPD prefixes, i.e. almost all nodes need to have their score updated. So even if the optimal paths to all nodes in the MPD-DAG remain the same, the algorithm will still have to update all nodes that have one or more columns from the incoming alignment in their optimal path.

References

1. Holmes I, Durbin R: **Dynamic programming alignment accuracy**. *Journal of computational biology* 1998, **5**(3):493–504.

2. Dijkstra E: **A note on two problems in connexion with graphs**. *Numerische mathematik* 1959, **1**:269–271.

3. Gusfield D: **Algorithms on Stings, Trees, and Sequences: Computer Science and Computational Biology**. *ACM SIGACT News* 1997, **28**(4):41–60.

4. Hart P, Nilsson N, Raphael B: **A formal basis for the heuristic determination of minimum cost paths**. *IEEE transactions on Systems Science and Cybernetics* 1968, **4**(2):100–107.
